# Supplementary material for: Reliability of heart rate and respiration rate measurements with a wireless accelerometer in postbariatric recovery
Source: PLoS One. 2021 Apr 28;16(4):e0247903. doi: 10.1371/journal.pone.0247903 (PMC8081266; doi:10.1371/journal.pone.0247903)
Supplement: S1 Table — Statistics of the HeartR vitals per patient on a 1-sec-period. The mean differences are shown in the first column, the CIs are shown in columns 2 and 3. The gray values are the values which exceed the threshold of 5 bpm. (PDF) [file pone.0247903.s001.pdf]

# Patient	Mean differences Patient monitor vs. Healthdot	CI (mean + SD*1.96)	CI (mean-SD*1.96)
1	-7,65	36,22	-51,51
2	0,68	4,22	-2,87
3	0,23	1,94	-1,48
4	0,79	7,79	-6,21
5	-0,34	4,45	-5,14
6	2,62	13,19	-7,95
7	0,45	2,25	-1,35
8	0,19	2,84	-2,45
9	-26,32	34,56	-87,19
10	0,54	5,77	-4,68
11	1,19	8,74	-6,37
12	0,59	3,72	-2,54
13	-40,24	32,68	-113,16
14	0,03	1,65	-1,58
15	0,93	6,81	-4,95
16	1,37	7,62	-4,88
17	0,73	4,73	-3,26
18	-0,09	3,57	-3,76
19	0,32	3,47	-2,83
20	-0,24	2,43	-2,90
21	-0,40	4,24	-5,03
22	0,23	4,56	-4,10
23	1,12	6,34	-4,09
24	0,03	4,69	-4,62
