# Supplementary material for: Reliability of heart rate and respiration rate measurements with a wireless accelerometer in postbariatric recovery
Source: PLoS One. 2021 Apr 28;16(4):e0247903. doi: 10.1371/journal.pone.0247903 (PMC8081266; doi:10.1371/journal.pone.0247903)
Supplement: S2 Table — Statistics of the HeartR vitals per patient on a 5-min- average. The mean differences are shown in the first column, the CIs are shown in columns 2 and 3. The gray values are the values which exceed the threshold of 5 bpm. (PDF) [file pone.0247903.s002.pdf]

# Patient	Mean differences Patient monitor vs. Healthdot	CI (mean + SD*1.96)	CI (mean-SD*1.96)
1	-11,62	27,32	-50,56
2	0,66	1,41	-0,09
3	0,17	0,47	-0,12
4	0,24	1,11	-0,63
5	-0,19	0,12	-0,49
6	NaN	NaN	NaN
7	0,43	0,56	0,29
8	0,18	0,36	-0,01
9	NaN	NaN	NaN
10	0,25	0,80	-0,31
11	0,78	1,85	-0,29
12	0,58	0,81	0,35
13	-46,81	31,24	-124,86
14	-0,02	0,19	-0,22
15	0,52	1,21	-0,18
16	NaN	NaN	NaN
17	0,84	1,25	0,43
18	-0,16	0,59	-0,92
19	0,30	0,60	0,00
20	-0,25	-0,07	-0,43
21	-0,37	0,49	-1,23
22	-0,14	*NA*	*NA*
23	1,18	1,55	0,81
24	0,01	0,29	-0,28
