# Supplementary material for: Reliability of heart rate and respiration rate measurements with a wireless accelerometer in postbariatric recovery
Source: PLoS One. 2021 Apr 28;16(4):e0247903. doi: 10.1371/journal.pone.0247903 (PMC8081266; doi:10.1371/journal.pone.0247903)
Supplement: S3 Table — Statistics of the RespR vitals per patient on a 1-sec-period. The mean differences are shown in the first column, the CIs are shown in columns 2 and 3. The gray values are the values which exceed the threshold of 5 rpm. (PDF) [file pone.0247903.s003.pdf]

# Patient	Mean differences Patient monitor vs. Healthdot	CI (mean + SD*1.96)	CI (mean-SD*1.96)
1	1,10	8,37	-6,16
2	0,44	3,53	-2,65
3	0,59	5,76	-4,57
4	-0,21	5,24	-5,66
5	1,35	9,03	-6,33
6	0,11	6,62	-6,41
7	5,69	13,89	-2,51
8	0,60	3,57	-2,37
9	-1,34	1,81	-4,48
10	0,76	6,15	-4,64
11	-0,36	6,36	-7,08
12	0,40	4,20	-3,39
13	2,30	9,14	-4,54
14	4,00	13,66	-5,66
15	0,13	6,04	-5,77
16	5,57	17,39	-6,25
17	1,57	4,41	-1,28
18	1,96	8,11	-4,20
19	1,68	6,75	-3,39
20	0,92	3,69	-1,85
21	1,41	6,81	-3,98
22	1,00	4,10	-2,11
23	0,75	4,46	-2,97
24	1,49	7,13	-4,15
25	0,46	4,71	-3,79
26	2,66	10,21	-4,90
