# Supplementary material for: Reliability of heart rate and respiration rate measurements with a wireless accelerometer in postbariatric recovery
Source: PLoS One. 2021 Apr 28;16(4):e0247903. doi: 10.1371/journal.pone.0247903 (PMC8081266; doi:10.1371/journal.pone.0247903)
Supplement: S4 Table — Statistics of the RespR vitals per patient on a 5-min- average. The mean differences are shown in the first column, the CIs are shown in columns 2 and 3. The gray values are the values which exceed the threshold of 5 rpm. (PDF) [file pone.0247903.s004.pdf]

# Patient	Mean differences Patient monitor vs. Healthdot	CI (mean + SD*1.96)	CI (mean-SD*1.96)
1	-0,29	2,78	-3,36
2	0,33	1,60	-0,94
3	0,09	1,09	-0,90
4	NaN	NaN	NaN
5	-0,01	1,25	-1,28
6	0,60	1,34	-0,14
7	NaN	NaN	NaN
8	0,58	1,98	-0,81
9	-1,27	0,08	-2,63
10	NaN	NaN	NaN
11	-0,16	1,40	-1,72
12	0,03	1,17	-1,11
13	2,23	5,57	-1,11
14	2,48	8,14	-3,19
15	-0,40	1,54	-2,33
16	7,03	12,55	1,51
17	NaN	NaN	NaN
18	1,52	3,18	-0,14
19	1,47	3,80	-0,86
20	0,90	2,22	-0,42
21	1,14	3,81	-1,54
22	1,12	2,27	-0,03
23	0,46	NaN	NaN
24	NaN	NaN	NaN
25	0,09	1,17	-1,00
26	2,24	5,40	-0,93
