# Supplementary material for: Reliability of heart rate and respiration rate measurements with a wireless accelerometer in postbariatric recovery
Source: PLoS One. 2021 Apr 28;16(4):e0247903. doi: 10.1371/journal.pone.0247903 (PMC8081266; doi:10.1371/journal.pone.0247903)
Supplement: S1 Study protocol — (PDF) [file pone.0247903.s005.pdf]

Clinical Study Investigation Plan
PJ-013483 FLAGSHIP Transitional Care Study 3

This Clinical Study* protocol template is for use with ICBE studies formerly known as H, H-Lite, J, J-Lite. These studies undergo full ICBE Board review (not offline review). This template is ISO 14155 compliant; however, not all clinical studies will follow ISO 14155 and thus non-applicable elements can be noted as such.

*A systematic investigation in one or more human subjects, undertaken to assess the safety or performance of a medical device (ISO 14155). NOTES: 1. “Clinical trial” and “clinical study” are synonymous with “clinical investigation”; 2. The Netherlands *WMO* defines a “clinical trial” as medical research and in which persons are subjected to treatment or are required to follow a certain behavioral strategy.

You must undergo a Regulatory Intake with the Director of Q&R before you submit your study to ICBE for review. This ensures your study considers potentially relevant matters such as REC review, database registration, prototype release/DOC, competent authority notification, etc.

**Document history**			
Version:	Date:	Author:	Description/Change/Reason for Change
0.1	*2018-Sep-03*	*Joerg Liebmann*	*Initial version*
0.2	*2018-Sep-20*	*Helma de Morree*	*Inserted ELAN specific passages*
0.3	*2018-Oct-11*	*Helma de Morree / Joerg Liebmann*	*Inserted reviewer comments*
0.4	*2018-Oct-25*	*Helma de Morree / Joerg Liebmann*	*Addressed coach comments and comments by privacy officer*
0.5	*2018-Nov-22*	*Helma de Morree / Joerg Liebmann*	*Adressed comments by reviewers*
1.0	*2018-NOV-26*	*Helma de Morree / Joerg Liebmann*	*APPROVED by ICBE*
1.1	*2019-JAN-31*	*Helma de Morree / Joerg Liebmann*	*Implemented changes requested by METC*
2.0	*2019-FEB-05*	*Helma de Morree / Joerg Liebmann*	*APPROVED by ICBE*
2.1	2019-SEP-27	*Helma de Morree / Joerg Liebmann*	Amendment 1 after METC approval due to study prolongation and interim analysis (see Reason for amendment_PJ-013483_FLAGSHIP-Transitional-Care-Study-3 for a detailed description of all changes including a justification)
2.2	2019-NOV-01	*Helma de Morree / Joerg Liebmann*	Added additional baseline population sample
2.3	2019-NOV-15	*Helma de Morree / Joerg Liebmann*	Comments from reviewers processed
2.4	2019-NOV-27	*Helma de Morree / Joerg Liebmann*	Comments from ICBE processed
3.0	2019-NOV-29	*Helma de Morree / Joerg Liebmann*	Approved by ICBE

Philips Research

PHILIPS

APPROVAL / PROTOCOL SIGNATURE SHEET

Function	Name	Signature /Date (yyyy-Mmm-dd)
Head of Department	Johannes Nürnberg (Department Head PC&M, Philips)	2019.12.05
Investigator	Arthur Bouwman	2019-12-09
Principal Investigator	Eveline Mestrom	i.a. 2019-12-11
Investigator	Simon Nienhuijs	
Investigator	Ignace de Hingh	10/12/2019
Investigator	Volkher Scharnhorst	9/12/19
Investigator	Jai Scheerhoorn	2019-Dec-05
Investigator	Jonna van der Stam	2019-12-9
	**Additional/other**	
Author	Joerg Liebmann	2019-Dec-04
Author	Helma de Morree	2019-Dec-05
Reviewer	Reinder Haakma	5/12/2019
Reviewer	Timon Grob	2019-Dec-06
Reviewer	Michael Heesemans	2019-Dec-05

APPROVAL / PROTOCOL SIGNATURE SHEET

Function	Name	Signature /Date (yyyy-Mmm-dd)
**Head of Department**	Johannes Nürnberg (Department Head PC&M, Philips)	
**Investigator**	Arthur Bouwman	
**Principal Investigator**	Eveline Mestrom	
**Investigator**	Simon Nienhuijs	2019-DEC-04
**Investigator**	Ignace de Hingh	
**Investigator**	Volkher Scharnhorst	
**Investigator**	Jai Scheerhoorn	
**Investigator**	Jonna van der Stam	
	**Additional/other**	
**Author**	Joerg Liebmann	
**Author**	Helma de Morree	
**Reviewer**	Reinder Haakma	
**Reviewer**	Timon Grob	
**Reviewer**	Michael Heesemans	

ROLES / RESPONSIBILITIES

This section contains a list of all parties and persons involved in the Clinical Investigation.

**Principal Investigator(s)**	Drs. Eveline Mestrom (eveline.mestrom@catharinaziekenhuis.nl)<br>+31 (0)40 - 239 85 00
**Investigator(s)**	Dr. Arthur Bouwman, (arthur.bouwman@catharinaziekenhuis.nl)<br>Dr. Simon Nienhuijs (simon.nienhuijs@catharinaziekenhuis.nl)<br>Dr. Ignace de Hingh (ignace.d.hingh@catharinaziekenhuis.nl)<br>Dr. Volkher Scharnhorst (volkher.scharnhorst@catharinaziekenhuis.nl)<br>Drs. Jai Scheerhoorn (jai.scheerhoorn@catharinaziekenhuis.nl)<br>Ir. Jonna van der Stam (jonna.vd.stam@catharinaziekenhuis.nl)
**Investigational Site**	Department of Anesthesiology and Department of Surgery, Catharina Hospital, Michelangelolaan 2, 5623 EJ Eindhoven, The Netherlands
**Other participating sites**	N/A
**Sponsor**	Philips Electronics Nederland BV, acting through Research, Eindhoven, NL
**Subsidizing party**	N/A
**Independent physician(s) (the Netherlands only)**	Ashley J R De Bie<br>Internal Medicine and Intensive Care; Catharina Hospital, Michelangelolaan 2, 5623 EJ Eindhoven, The Netherlands
**Laboratory sites**	N/A
**Other / Appendix**	
Clinical study lead Philips:Joerg Liebmann, joerg.liebmann@philips.com; Helma de Morree helma.de.morree@philips.com	

TABLE OF CONTENTS

APPROVAL / PROTOCOL SIGNATURE SHEET 2
TABLE OF CONTENTS 4
LIST OF ABBREVIATIONS AND RELEVANT DEFINITIONS 5
INTRODUCTION 7
1 EXECUTIVE SUMMARY 8
2 INVESTIGATIONAL DEVICE 14
2.1 Device Summary 14
2.1.1 HealthDot: 14
2.1.2 Elan: 15
2.2 Intended use 17
2.2.1 HealthDot: 17
2.2.2 Elan: 17
2.3 Device Description 18
2.3.1 HealthDot: 18
2.3.2 Elan 20
3 JUSTIFICATION 21
4 RISKS AND BENEFITS ASSESSMENT 23
5 OBJECTIVES AND HYPOTHESES 24
6 CLINICAL INVESTIGATION DESIGN 25
6. 1 General 25
6.2 Investigational device(s) and comparator(s) 26
6.3 Subjects 26
6.4 Procedures 28
Specimen Banking (saving human fluid or tissue for future research) 28
6.4.1 Screening (Visit 0) 28
6.4.2 Prebaseline visit (only 20 patients) 28
6.4.3 Start (Baseline visit) 28
6.4.4 Discharge from hospital 28
6.4.5 Follow up 29

6.5 Monitoring plan 29
7 STATISTICAL CONSIDERATIONS 29
8 DATA MANAGEMENT 30
9 AMENDMENTS 32
10 DEVIATIONS 32
11 DEVICE ACCOUNTABILITY 33
12 STATEMENTS OF COMPLIANCE 33
13 INFORMED CONSENT PROCESS 34
14 ADVERSE EVENTS AND DEVICE DEFICIENCIES 35
15 INVESTIGATOR BROCHURE (IB) 37
16 VULNERABLE POPULATION 38
17 SUSPENSION OR PREMATURE TERMINATION 38
18 PUBLICATION POLICY 39
19 BIBLIOGRAPHY 40
20 APPENDIX I 40
21 APPENDIX II 42

LIST OF ABBREVIATIONS AND RELEVANT DEFINITIONS

ABR	ABR form, General Assessment and Registration form, is the application form required for submission to the accredited Ethics Committee<br>*In Dutch, ABR = Algemene Beoordeling en Registratie*
AE	Adverse Event<br>Any untoward medical occurrence, unintended disease or injury, or untoward clinical signs (including abnormal laboratory findings) in subjects, users or other persons, whether or not related to the investigational medical device
AR	Adverse Reaction
CA	Competent Authority
CCMO	Central Committee on Research Involving Human Subjects<br>*In Dutch, CCMO = Centrale Commissie Mensgebonden Onderzoek*
Clinical Investigation	Systematic investigation in one or more subjects, undertaken to assess the safety and performance of a medical device
CIP	Clinical Investigation Plan<br>Document that state(s) the rationale, objectives, design and proposed analysis, methodology, monitoring, conduct and record-keeping of the clinical investigation<br>NOTE: The term “protocol” is synonymous with “CIP”. However, protocol has many different meanings, some not related to clinical investigation, and these can differ from country to country.
CRF	Case Report Form<br>Set of printed, optical or electronic documents for each subject on which information to be reported to the sponsor is recorded, as required by the CIP
CV	Curriculum Vitae
DSMB	Data Safety Monitoring Board
Endpoint	Principal indicator(s) used for assessing the primary hypothesis of a clinical investigation

EU	European Union
EWS	early warning scoring
GCP	Good Clinical Practice
IB	Investigator's Brochure
IC	Informed Consent<br>The informed consent is documented by means of a written, signed and dated informed consent form.<br>The informed consent process is the process by which an individual is provided information and is asked to voluntarily participate in a clinical investigation.
Investigation site	Institution or site where the clinical investigation is carried out
IRB	Institutional Review Board
HIPEC	Hyperthermic intraperitoneal chemotherapy
Hypothesis	Testable statement, resulting from the objective, regarding the investigational medical device safety or performance used to design the clinical investigation and that can be accepted or rejected based on results of the clinical investigation and statistical calculations.<br>NOTE: The primary hypothesis is the determinant of the investigational medical device safety or performance parameters and is usually used to calculate the sample size. Secondary hypotheses concerning other points of interest can also be evaluated.
Investigator	Individual member of the investigation site team designated and supervised by the principal investigator at an investigation site to perform critical clinical-investigation-related procedures or to make important clinical investigation- related decisions.<br>NOTE: An individual member of the investigation site team can also be called "sub-investigator" or "co-investigator".
METC	Medical Research Ethics Committee (MREC)<br>*In Dutch, METC = Medisch Ethische Toetsing Commissie*
Objective	Main purpose for conducting the clinical investigation
Point of enrollment	Time at which, following recruitment, a subject signs and dates the informed consent form
SAE	Serious Adverse Event<br>Adverse event that<br>a) led to death,<br>b) led to serious deterioration in the health of the subject, that either resulted in<br>1) a life-threatening illness or injury, or<br>2) a permanent impairment of a body structure or a body function, or<br>3) in-patient or prolonged hospitalization, or<br>4) medical or surgical intervention to prevent life-threatening illness or injury or permanent impairment to a body structure or a body function,<br>c) led to foetal distress, foetal death or a congenital abnormality or birth defect<br>NOTE Planned hospitalization for a pre-existing condition, or a procedure required by the CIP, without serious deterioration in health, is not considered a serious adverse event.
Sponsor	Individual or organization taking responsibility and liability for the initiation or implementation of a clinical investigation
SFU	Step forward unit
WBP	Personal Data Protection Act (in Dutch: Wet Bescherming Persoonsgevens)
WMO	Medical Research Involving Human Subjects Act<br>*In Dutch, WMO = Wet Medisch-wetenschappelijk Onderzoek met Mensen*

INTRODUCTION

Postoperative complications are frequently encountered at the surgical ward. Complications can lead to major adverse events such as unplanned intensive care unit (ICU) admissions, cardiorespiratory arrest or even death with a postoperative in-hospital mortality rate of 4%. They are also associated with prolonged length of stay. [1] Timely detection of patients at risk of complications is important to initiate appropriate treatment and prevent major adverse events.

Various strategies have been developed and studied to reduce the number of adverse events. [2,3] Risk stratification is applied in the pre- and intra-operative setting by taking into account patient characteristics and vital signs. These vital signs are collected once during pre-operative screening and continuously during surgery. Postoperatively, there are roughly two options. For low or intermediate surgery risk, the patient is transferred to the recovery room where continuous monitoring is maintained until the patient is deemed hemodynamically stable and transferred to the ward. The other option is admission to the ICU where intensive monitoring is continued for an appropriate amount of time, before the patient is transferred to the ward. Once at the ward, less frequent assessments of vital signs and laboratory values take place. Early warning scores (EWS) have been developed as an objective bedside tool to help clinicians and nurses to identify patients at risk of adverse events by using vital signs. To assist completion of the EWS, electronic automated systems have been introduced since several studies emphasized that these systems provide a faster completion of EWS with increased accuracy. [2,3] This is important for the effectiveness of EWS since complete registrations on a regular basis are necessary and upcoming assisting medical technologies, like clinical decision support tools and prediction models, rely on these data and their accuracy and complete documentation. Track and trigger systems have shown potential in early deterioration detection by analyzing periodic measurements and automatically alert medical staff when a predetermined threshold is reached.

However, the abovementioned strategies have limitations. First, data of the different monitoring locations is collected separately in the electronic medical record (EMR). This results in less integration of knowledge throughout the perioperative journey. Second, literature provides a large variability of studies on monitoring systems and track and trigger systems with little evidence of reliability, validity and utility. Study populations are mostly selected on general hospital wards, whereas the surgical patients are at increased risk of complications. Third, to establish EWS in a practical way, the data is calculated using dichotomous instead of continuous data. Studies using continuous data have shown better prediction algorithms for deterioration. Also, mostly vital parameters are taken into account, while medication and laboratory values have shown to be relevant predictors as well. Fourth, machine learning is useful for prediction and clinical decision support. However, input is required from reliable continuous monitoring such as wearable sensors, which are preferentially well tolerated by patients. Finally, there is scarce data on the value of preoperative data to provide more individual context or a personalized baseline for vital parameters. Heart rate and respiratory rate were found to be abnormal in postoperative patients even without development of complications.[4] However, the deviation from baseline was not examined and could provide more information on the trend in postoperative recovery on an individual basis. The influence of, for example, preoperative quality of sleep or subjective feelings of anxiety or the gut feeling of relatives on outcome is not known yet.

Concluding, the technology is available but not mature enough yet to be implemented on a large scale, and the predictive value of home monitoring has not been studied yet. Our long-term aim is to create a care continuum by collecting and combining data throughout the perioperative journey into integrated patient-specific risk assessment and warning for post-operative deterioration. For this

purpose, the current study will test i.a. accelerometry and photoplethysmography (PPG) measurement technologies in real life and assess the usability issues and opportunities.

1 EXECUTIVE SUMMARY

**Study Title & ID**	PJ-013483 FLAGSHIP Transitional care study 3 [TRICA]
**Project Name**	PJ-013483 Intellivue discovery
**Brief Summary of Study**<br>***Provide a lay-language abstract of your study plan (100-300 words)*** *https://humansubjects.stanford.edu/new/docs/glossary_definitions/lay_language.pdf*	In this study patients with elective surgery will wear two devices (HealthDot and Elan) after surgery in hospital and after discharge at home for up to 2 weeks (HealthDot) or 3 weeks (Elan). Additionally, 20 patients of the total of 350 planned patients, will be recruited to wear the HealthDot (for up to 1 week) as well as the Elan (for 48 hours) before surgery to get their baseline values on heart rate and respiratory rate. The HealthDot will measure breast motion by accelerometer and calculate heart rate, posture, activity and respiratory rate which are stored on the device as well as sent via LoRa network to Philips. The Elan device will measure PPG and accelerometer data which is transferred via an MSX (Monitoring Study boX) to Philips. The data collected will be used for algorithm development. Data will be analysed retrospectively and compared to readmission and adverse events to see if the events could have been predicted due to the collected data by the devices. No clinical decisions will be based on the measurements done during the study.
**Key Words Associated with Study**<br>**Provide 3-5 PubMed MeSH keywords (https://www.ncbi.nlm.nih.gov/mesh)**	Heart rate, Respiratory rate, Activity, Clinical Deterioration, Major Surgery, Bariatric Surgery
**Country(s) from where the data originates (where collected)**	☒ Netherlands ☐ USA ☐ Germany ☐ China<br>☐ India ☐ Belgium ☐ UK ☐ Kenya<br>☐ Other:
**ISO 14155 compliance**	☐ This study will be conducted per the requirements of ISO 14155<br>☒ This study WILL NOT be conducted per the requirements of ISO 14155. For example studies formerly known as H-lite and J-Lite. These devices are not medical devices according to the definition of Chapter 1, Article 2 paragraph 1 of the Regulation (EU) 2017/745 on medical devices (MDR) because they are not intended by the manufacturer to be used to diagnose, prevent,

	monitor, predict, prognose, treat or alleviate a disease, diagnose, monitor, treat, alleviate or compensate for an injury or disability or investigate, replace or modify the anatomy or a physiological or pathological process or state. All data collected by the device will be analysed retrospectively only and no clinical action or decision will be based on these data. These data will not be used in the care giving process or derive medical claims. Therefore, full compliance with ISO 14155 is not needed.
**Is this study part of a student project/thesis?**	☒ No<br>☐ Yes, Student Name: University:
**Primary objective (only one)**	To evaluate the sensitivity and specificity for the prediction of deterioration after surgery using the data calculated based on accelerometer and/or PPG measurements.
**Secondary objective(s)**	• To evaluate if the calculated heart rate and respiratory rate data are accurate compared to a gold standard as used in the hospital.<br>• To evaluate the usability of the devices from a hospital staff perspective not interfering with hospital workflow.<br>• To evaluate the usability of the devices from a patient perspective not interfering with normal daily activities.<br>• To evaluate if connectivity is good during the patient journey and geolocation can be assessed.<br>• To technically validate offline metrics using ELAN collected data in the perioperative period as input (PPG and ACC) compared to those obtained from gold standard patient monitoring, which includes ECG (for heart rate), pulse oximetry, body temperature and invasive or non-invasive blood pressure measurements as part of standard care.
**Participating parties and their roles**	This study is sponsored by Philips Electronics Nederland BV, acting through Research, Eindhoven, NL. The legal manufacturer of the experimental prototype HealthDot is Philips Electronics Nederland BV, acting through Research, Eindhoven, NL.<br><br>The legal manufacturer of the Elan is Philips Electronics Nederland BV.<br><br>The study will be conducted at the Department of Anesthesiology, Catharina Hospital in Eindhoven by dr. R.A. Bouwman and drs. E. Mestrom as investigators.
**Subjects**	350 patients, scheduled for surgery e.g. bariatric and major surgery such as cytoreductive surgery and hyperthermic intraperitoneal chemotherapy (HIPEC), complex rectal surgery, esophagectomy and pancreatectomy.<br><u>Inclusion criteria:</u><br>• Adult<br>• Willing and able to sign informed consent form

	• Willingness to abstain from visiting a sauna during the study period<br>• Willingness to dry area where the HealthDot is applied in a dipping fashion after washing<br>• Willingness to abstain from flying during the study period of time<br>• Elective surgery<br>• General anesthesia required for surgery<br><br>Exclusion criteria:<br>• General inmates of psychiatric wards, prisons, or other state institutions<br>• Investigator or any other team member involved directly or indirectly in the conduct of the clinical study<br>• Any skin condition, for example prior rash, discoloration, scars or open wounds at the area of investigation of both devices<br>• Pregnant, or breastfeeding<br>• Known to be allergic for the tissue adhesive used in the HealthDot.<br>• Use of topical that is known to influence the skin at the test area (such as medical and non-medical creams or lotions)<br>• Patient with active implantables such as Implantable Cardioverter Defibrilator (ICD) and pacemaker<br>• Unable to understand instructions<br>• Expected participation less than 2 weeks<br>• Left lower rib (place where HealthDot will be applied) is involved in the area of surgery, area of disinfection or area where bandages are needed.<br>• Area on arm where the Elan device is applied is involved in the surgical procedure.<br>• Patients with antibiotic resitant infections (e.g. MRSA).
**Investigation design**	This is a study at one site in the Netherlands. No randomization or blinding will be done.
**Investigation procedures**	The HealthDot and Elan devices will be applied directly after surgery and patients (n=350) will wear the devices for a total time of 14 days (HealthDot) and 21 days (Elan) including time after discharge from the hospital. The surgical procedure is not part of the study described here. For some of these patients it is planned to compare the measurements of both devices with the hospital vital signs monitor regarding heart rate and respiratory rate when these data are available. This will be done for patients who receive standard monitoring after surgery. Additionally, 20 patients of the total of 350 planned patients, will be recruited to wear the HealthDot (for up to 1 week) as well as the Elan (for 48 hours)

	before surgery to get their baseline values on heart rate and respiratory rate.
**At-Home Subject Procedures**	Subjects are expected to wear the HealthDot investigated in this study for up to 14 days at home. Additionally, they are expected to fill in a daily diary as well as a simple questionnaire at the end of the study. For the 20 patients who will receive the Healthdot for baseline measurements this study procedure is prolonged by wearing the Healthdot 7 days before entering the hospital for surgery.<br><br>Subjects will also be asked to wear the Elan on their non-dominant wrist for up to 21 days at home. They will need to offload and recharge the Elan device every night (while using a second Elan device) with the MSX device provided together with the Elans. For the 20 patients who will receive the Elan for baseline measurements this study procedure is prolonged by wearing the Elan for 2 times 24 hours (2 days overall) before entering the hospital for surgery.
**Devices**<br><br>**Check each box as appropriate and add a brief description of the device(s)—e.g., fitness tracker, VR goggles, breast pump, electric toothbrush, ELAN, MRI scanner.**<br><br>**Devices will be described fully in Section 2.1 of the study protocol.**	☐ None<br>☐ Mock-up (see ICBE FAQ definition):<br>☒ Survey/questionnaire (if copyrighted, ensure permission for use):<br>☐ Interview/focus group with audio/video taping**:<br>☐ Interview/focus group without audio/video taping**:<br>**see decision flowchart regarding interviews vs QI vs consulting https://share-intra.philips.com/sites/STS20131115093003/ICBE-FAQ/Lists/Photos/interviews%20criteria%20flowchart%20feb2018.pptx<br>☐ FDA or CE-approved medical device within intended use:<br>☐ FDA or CE-approved medical device outside intended use:<br>☒ FDA or CE-approved non-medical device within intended use: Elan<br>☐ FDA or CE-approved non-medical device outside intended use:<br>☐ In vitro medical device:<br>☐ Medical device prototype***:<br>☒ Non-medical device prototype***: HealthDot, MSX<br>☐ Software prototype***, non-medical:<br>☐ Software prototype***, medical:<br>☐ Other:<br><br>***Before a study employing prototypes may start, a Declaration of Conformity (DoC) needs to be issued by the Director Regulatory Affairs. With this DoC, Philips declares that the device can safely be used in the study. It may be needed to work according to some formal standards to build up this evidence (e.g. with respect to bio-compatibility or sterility).<br><br>***☒ DoC needed (brief overview of the actions that will be taken to get the Declaration of Conformity. See Philips Research QMS procedure “Procedure Product Release (QR-PRO-36”. Give details of how the project will deal with the regulatory requirements. Consult the Q&R office when needed).

**Milestones**	First patient first visit: April 2019<br><br>Last patient last visit: June 2020<br><br>Duration: 15 months<br><br>Individual subject participation is up to 3 weeks including time in hospital and at home. For 20 subjects wearing the devices before surgery for baseline measurement the time of participation will prolong by one week to up to 4 weeks (depending on surgery schedule).
**Claims**	Will you obtain regulatory (FDA, CE) approval for a new product or service, a new indication, or a new marketing claim?<br>☐ Yes ☒ No<br><br>If YES, in what countries will the claims be registered?<br>☐ Netherlands ☐ USA ☐ Germany ☐ China<br>☐ India ☐ Belgium ☐ UK ☐ Kenya<br>☐ Other:<br><br>Have you developed a list of claims that you would like to assert for your product or service? ☐ Yes ☒ No
**External Research Ethics Committee (REC) Approval**<br>**Note: if your document is not in English or Dutch, include an English translation**	☐ Not Required (not a local/state/country requirement)<br>☒ Required (apply for it AFTER ICBE approval)<br>☐ Approval already obtained (upload for review by ICBE)<br>☐ REC Review is waived (upload review waiver)<br>☐ REC has waived requirement for informed consent (upload waiver)<br>☐ Check this box if more than one REC is involved in this study and identify them:
**Submission to Competent Authorities**	☒ Not applicable<br><br>Check the applicable option concerning Competent Authority approval for medical device studies:<br>☐ No submission to Competent Authority needed, since no non-released medical device involved<br>☐ Approval from Competent Authority is mandatory for this study with a non-released medical device<br><br>Note: Released medical device means a device that is e.g. CE-marked (93/42/EEC) if study takes place in EU, or has 510k if study takes place in USA.
**Database registration**	☐ None.<br>☒ ClinicalTrials.gov<br>☒ Other: Nederlands Trial Register
**Type of Legal Agreement for this study**	☐ None required ☐ MRA Exhibit<br>☒ Contract ☐ Purchase Order
**Status of Legal Agreement**	☐ NA ☐ Not started ☐ In Process ☒ In Place<br>Name of Legal Rep: Ciska van Kleef; David Stretton<br>This study runs under the Flagship contract which is already in place. A separate data transfer agreement has been setup and signed.

**IP Strategy**	The IP strategy is described in detail in the existing contract Impuls 2 HC Flagship topic Peri-operative_Final_extended_signed-by_all
**ICBE Training is required for all key study personnel**	☒ YES, this training is completed as required by ICBE*<br>☐ NO, this training is not completed as required by ICBE*:<br>*See FAQ: https://share-intra.philips.com/sites/STS20131115093003/ICBE-FAQ/Lists/Posts/Post.aspx?ID=159

2 INVESTIGATIONAL DEVICE

2.1 Device Summary

This chapter contains a summary description of the **investigational devices** and their intended purpose.

2.1.1 HealthDot:

**Summary device description**	The HealthDot is a prototype of a wearable data logger. It consists of an adhesive layer, electronics and a battery. It contains an accelerometer which measures movement including movement of the lung and heart, processes these movement data with a validated algorithm to calculate heart rate, respiratory rate, posture and activity which are stored on the device and send out through the LoRa network. This can be a local gateway and/or gateways provided by KPN. The total system to be tested consists of:<br>1. HealthDot<br>2. Gateway<br>3. KPN-LoRa network<br>4. Server<br>5. Dashboard<br>The system is an experimental non CE marked, non medical prototype which will get a Declaration of Conformity (DoC) provided by Philips before study starts.
**Summary intended use (for details see 2.2.1)**	The intended use of this system in this study is to gather movement data including movements of the heart and lung by means of an accelerometer during transitional care from in hospital to home for algorithm development. **All data collected will be analyzed retrospectively after the study has been closed only and no medical action or decision will be based on these data. These data will not be used in the care giving process or derive medical claims.**<br><br>Therefore, this device is not a medical device according to the definition of Chapter 1, Article 2 paragraph 1 of the Regulation (EU) 2017/745 on medical devices (MDR) because it is, as detailed above, not intended by the manufacturer to be used to diagnose, prevent, monitor, predict, prognose, treat or alleviate a disease, diagnose, monitor, treat, alleviate or compensate for an injury or disability or investigate, replace or modify the anatomy or a physiological or pathological process or state.
**Population description**	Patients with elective surgery that undergo transitions from hospital to home.
**Manufacturer**	Philips Electronics Nederland BV, acting through Research, Eindhoven, NL
**Device model/type**	HealthDot v3.0
**Software version**	Revision 5
**Accessories**	N/A

**Traceability procedure**	The device traceability during and after the clinical investigation is documented in form “Device Accountability”. This record is intended to document the physical location of all investigational devices from shipment of investigational devices to the investigation sites until return or disposal.<br><br>The device is tracked to and from the investigational site by the Clinical study lead, while the trace to the subjects is done by the Principal Investigator. At clinical investigation termination or closure, device retrieval or disposure will be checked by the Monitor.<br><br>The devices are uniquely identified by a label containing a serial number (see device labeling).
**Required training**	The investigators will be instructed how to handle the HealthDot and where to position it. This will also be described in an instruction for use provided to the investigator. No specific training is required. The patient will also be informed on how to act and what to avoid while wearing the device with a separate instruction for use.
**Specific procedure acts**	N/A
**Device Labeling**	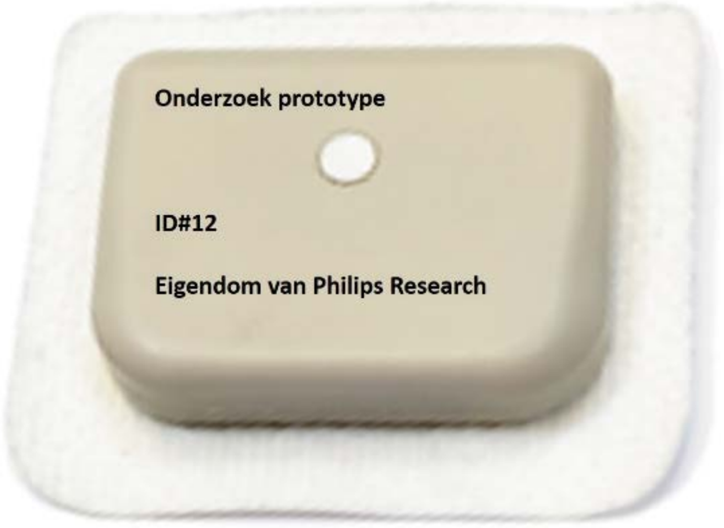

2.1.2 Elan:

**Summary device description**	The Elan system consists of:<br>• Elan<br>• MSX device<br>• Cloud storage<br>• Analytics engine<br><br>The Elan is a wrist-worn wearable (watch) that collects raw PPG and accelerometer data. The MSX device is a base station that automatically offloads the collected data from the wearable and uploads it to the cloud

	storage. An analytics engine then processes the data, extracts relevant metrics using previously developed algorithms and generates (patient) reports.<br><br>The Elan (Philips) is a CE-marked device, created for research purposes. The MSX device (Philips) is a non-CE marked research device. The security officer (Walter Belgers) has approved the system (Elan, MSX, cloud storage, analytics engine and web application) from a security standpoint (see documentation in the ICBE dossier).
**Summary intended use (for details see 2.2.2)**	The intended use of the Elan Device is to obtain and store reflective, green-spectrum photoplethysmography (PPG) data on intact skin of the human body. In addition to PPG data, also acceleration data (ACC) is collected using an internal 3D accelerometer. Logged data can be offloaded using a USB connection.
**Population description**	Patients with elective surgery that undergo transitions from hospital to home.
**Manufacturer**	Philips Electronics Nederland BV
**Device model/type**	Elan_CID_CE
**Software version**	2.2
**Accessories**	USB connector cable, MSX device
**Traceability procedure**	The device traceability during and after the clinical investigation is documented in form “Device Accountability”. This record is intended to document the physical location of all investigational devices from shipment of investigational devices to the investigation sites until return or disposal.<br><br>The device is tracked to and from the investigational site by the Clinical study lead, while the trace to the subjects is done by the Principal Investigator. At clinical investigation termination or closure, device retrieval or disposure will be checked by the Monitor.<br><br>The devices are uniquely identified by a label containing a serial number (see device labeling).
**Required training**	The investigators will be instructed how to handle the Elan and the MSX device. This will also be described in an instruction for use provided to the investigator. No specific training is required. The patient will also be informed on how to act and what to avoid while wearing the device with a separate instruction for use.
**Specific procedure acts**	N/A

**Device Labeling**	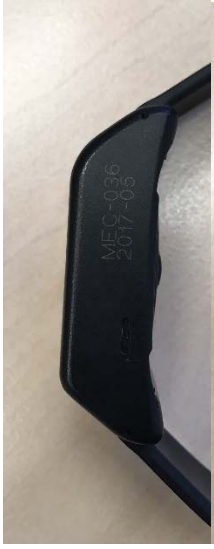 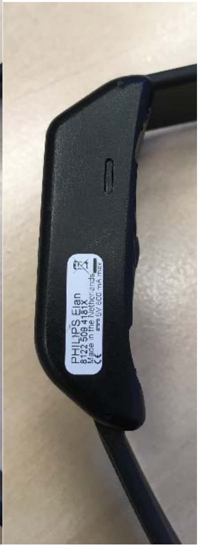

2.2 Intended use

2.2.1 HealthDot:

The intended use of this system in this study is to gather movement data including movements of the heart and lung by means of an accelerometer during transitional care from in hospital to home for algorithm development. For this we use an accelerometer containing research prototype called HealthDot which measures movement along the patient journey and which can calculating heart rate, respiratory rate, activity and posture based on these data and stores these data. These stored data will be sent via a wireless network to the sponsor. **All data collected will be analyzed retrospectively after the study has been closed only and no medical action or decision will be based on these data. These data will not be used in the care giving process or derive medical claims.**

Therefore, this device is not a medical device according to the definition of Chapter 1, Article 2 paragraph 1 of the Regulation (EU) 2017/745 on medical devices (MDR) because it is, as detailed above, not intended by the manufacturer to be used to diagnose, prevent, monitor, predict, prognose, treat or alleviate a disease, diagnose, monitor, treat, alleviate or compensate for an injury or disability or investigate, replace or modify the anatomy or a physiological or pathological process or state.

In this study, we will develop algorithms to evaluate whether we can retrospectively predict deterioration of surgical patients in hospital and at home based on the collected accelerometer data.

2.2.2 Elan:

The intended use of the Elan Device is to obtain and store reflective, green-spectrum photoplethysmography (PPG) data on intact skin of the human body. In addition to PPG data, also acceleration data is collected using an internal 3D accelerometer. Logged data can be offloaded using a USB connection.

During the study the data will only be checked for completeness and correctness. In case there are any problems with the data uploading, the researchers will contact the patient to solve the problem.

Patients will also be given contact details of the researchers to use in case of any technical problems or questions.

This device is not medical device according to the definition of Chapter 1, Article 2 paragraph 1 of the Regulation (EU) 2017/745 on medical devices (MDR) because they are not intended by the manufacturer to be used to diagnose, prevent, monitor, predict, prognose, treat or alleviate a disease, diagnose, monitor, treat, alleviate or compensate for an injury or disability or investigate, replace or modify the anatomy or a physiological or pathological process or state.

All data collected by the device will be analysed retrospectively only and no medical action or decision will be based on these data. These data will not be used in the care giving process or derive medical claims.

2.3 Device Description

2.3.1 HealthDot:

The total system to be tested consists of:

1. HealthDot
2. Gateway
3. KPN-LoRa network
4. Server
5. Dashboard (not implemented in hospital but at Sponsor site)

The HealthDot measures acceleration including movement of lung and heart, calculates heart rate, respiratory rate, activity and posture via a validated algorithm, and stores this on the device. Additionally, it sends these data out through the LoRa network to a secure Philips server.

A block diagram of the system is shown in **Figure 1:**

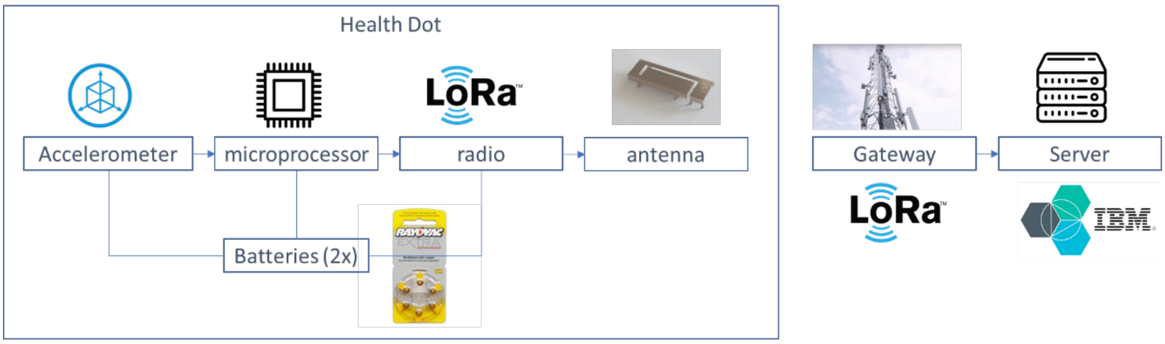

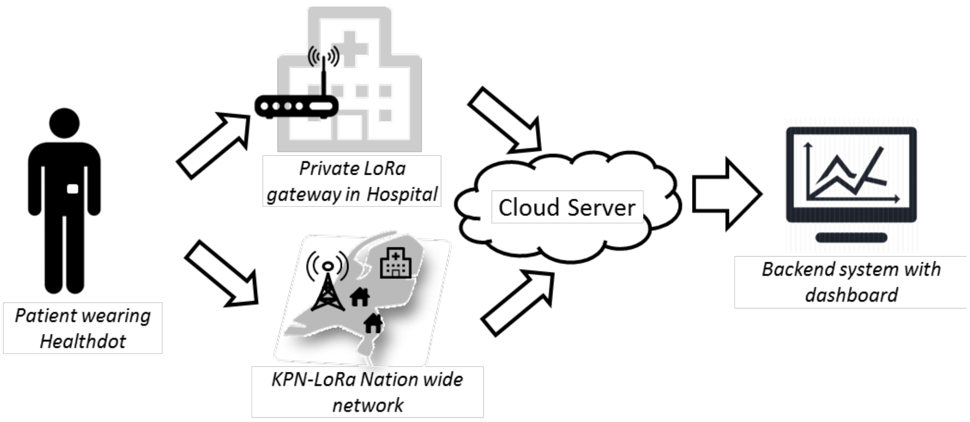

Figure 1, block diagram of HealthDot system and data flow

The HealthDot is a prototype of a wearable data logger. The HealthDot consists of an adhesive layer, electronics and a battery. The adhesive layer is a commercially available 4076 SC Spunlace Extended Wear Nonwoven Tape by 3M with a proven wear time of more than 14 days (external and internal tests). The electronic and battery are packaged in a waterproof housing. The adhesive layer (3M 4076) is skin compatible and has proven documentation that it can successfully be used for this pilot study. The housing (no skin contact) is made of biocompatible Terblend (Figure 2). The HealthDot can be removed by the patient themselves in case of emergency with a remover tissue which will be handed out to the patient during the baseline visit.

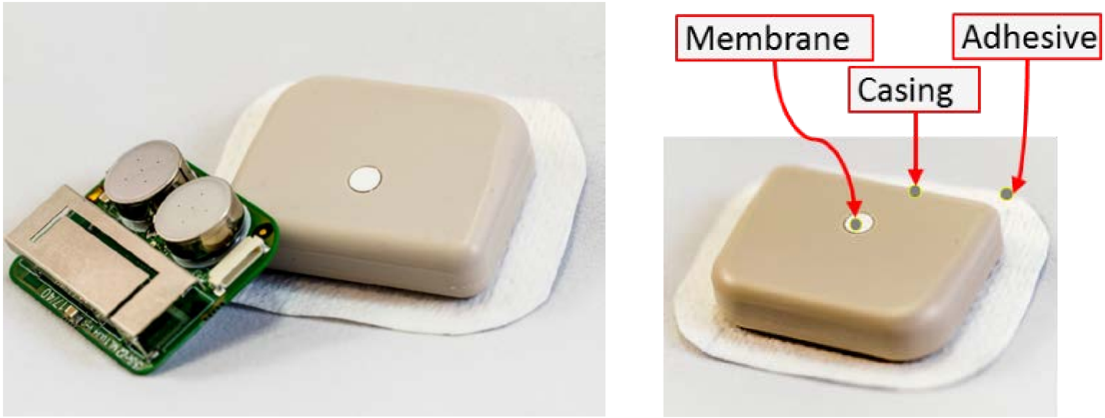

Figure 2. HealthDot, including adhesive.

Next to the HealthDot itself also gateways are needed. This can be a local gateway and/or gateways provided by KPN. For this trial a commercial gateway will be used from Multi-Tech Systems, Inc, with the product name: MTCAP-LEU1-868., an example is given in Figure 3. The declaration of conformity to the radio equipment directive (RED) can be found in Appendix II.

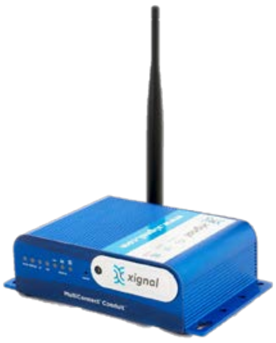

Figure 3. Example of commercial LoRa gateway.

Finally, a server (Health Suite Digital Platform, HSDP) is needed for collection of the information sent by the HealthDot via the gateways.

2.3.2 Elan

To be able to use the Elan for long-term recordings, it has been integrated in a system: the Apollo system. This system consists of:

- Elan
- Base station called MSX (Monitoring Study boX)
- Cloud storage
- Analytics engine

A schematic overview of the Apollo system is presented in Figure 4.

The Elan is a wrist-worn wearable (watch) that collects raw PPG and accelerometer data. The MSX is a device that automatically offloads the collected data from the wearable and uploads it to the cloud storage. An analytics engine then processes the data, extracts relevant metrics using previously developed algorithms and generates reports.

The Elan (Philips) is a CE-marked device, created for research purposes. The MSX (Philips) is a non-CE marked research device. The security officer (Walter Belgers) has approved the system (Elan, MSX, cloud storage, analytics engine and web application) from a security standpoint (see documentation in the ICBE dossier).

The strap of the Elan should not be worn too tight or too loose (as described in the user manual (IFU)). The investigator will take extra care while applying the wristband at the hospital and patients will be instructed accordingly. However, patients will have the option to loosen the strap by 1 hole in case of discomfort, to avoid them not wearing the wristband at all. The investigator at the hospital will make sure that the Elan is not placed on the same arm as a blood pressure cuff.

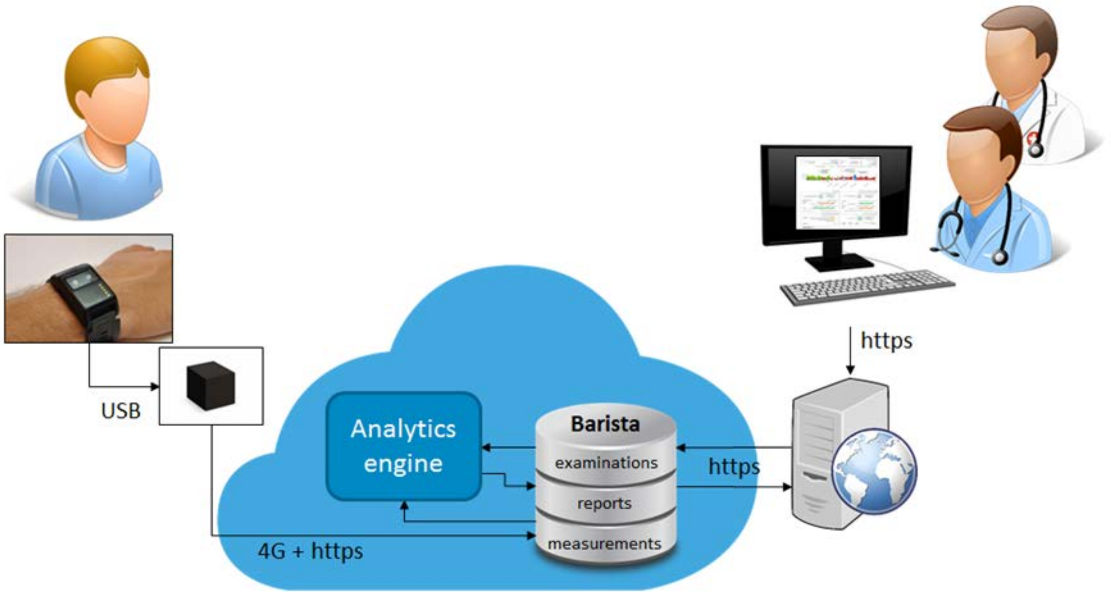

Figure 4. Schematic overview of the Apollo system.

3 JUSTIFICATION

The study is designed to evaluate whether the sensing methods are suitable for the purpose(s) and the population(s) for which they are intended.

The justification for the design of the study is based on the evaluation of pre-clinical data and the results of an assessment and analysis of risks concerning safety or performance of the HealthDot and Elan (see: Risk_Management_Summary_Matrix - FLAGSHIP Transitional care study 3 and Risk Management Plan - FLAGSHIP Transitional care study 3).

Since this is only a retrospective study not complying fully to ISO 14155, no formal but only an informal clinical literature review has been performed. The conclusion of this informal clinical literature review is that no valid data is available and therefore these data needs to be collected by this investigation detailed in this protocol.

This study is needed, because there is no scientific data available on the use of such sensing methods to measure movement data during the workflow of a patient going to hospital, being treated at the hospital and for the transition to home again. There is a strong need to test if the acquired data such as heart rate, respiratory rate, posture, sleep and activity in a real life setting can retrospectively predict deterioration of health post surgery at home. This cannot be done by bench testing.

This study adds information on the use of the sensing methods and this information is not available in literature.

The study needs to be performed on a population that fulfills the inclusion/exclusion criteria described in Chapter 6, defined by the principal investigator to mimic the real life workflow of the patient and to assess potential deterioration retrospectively during hospital stay and after leaving the hospital.

The inclusion of 20 subjects to wear the study devices also up to 1 week before surgery is necessary to see if a baseline measurement of heart rate and respiratory rate will be beneficial in assessing the endpoints. During and after surgery, deviation of vital parameters such as heart rate and respiratory rate takes place before postoperative recovery back to their baseline. Applying the devices after surgery only, has the disadvantage that the normal physiological parameters of the respective patient are not known.

Summary of Relevant Previous Clinical Experience

HealthDot:

A pilot study with healthy volunteers has been performed to assess wearability, connectivity and test the algorithm and wearability of the HealthDot to detect heart rate and breathing rate accurately. Therefore 25 persons wore the HealthDot on the lower left rib for 14 days. Full coverage with 3 private LoRa hubs was established on High Tech Campus, Eindhoven, in Building HTC31 (4 floors). Additionally, all 25 healthDots had in- and out- door access to the KPN LoRa nation wide network. So, a combination of private (local) gateway and a nation wide provider network (LoRa/KPN) was used to detect connectivity. The IBM Bluemix Cloud server was used during the trial to store data and results were displayed on a web based dashboard.

LoRa with private hubs:
Full coverage in HTC31 (4 floors) using 3 LoRa Hubs was achieved.
• It was not recorded when the volunteer left or entered the building.
• No package management was used (no synchronized sending, no confirmation, etc.)
LoRa with “Nation Wide” KPN network:
All volunteers were monitored via KPN throughout the Netherlands for 14 days (137,000 packets received)
• The analysis showed that the percentage of packages that arrive depending on the update interval were
– 75% of all packages arrived at every transmission (every 5 mins).
– 99% of all package updates times were within 45 mins.
– 99.99% of all package updates times were within 240 mins.
Algorithm performance - HR & RR of active healthy volunteers:
• The algorithm detected whether the patient was sufficiently at rest for a reliable HR and RR measurement. Measuring at rest is relevant for deterioration prediction.
• The HR and RR update intervals were highly depending on the daily activities (walking, washing, sporting, ..) of the volunteers.
Wearability and user comfort:
The HealthDot was evaluated on comfortability of the device to the volunteer. The overall comfort score after 14 days of wearing was between 8 and 9 on a scale of 0-10 whereas 10 is very comfortable and 0 is not comfortable.

Elan:
Studies have been done with the Elan where the Elan was worn for longer periods of time in healthy people and in patients. In the shift worker study, 110 healthy participants (36.6 +/- 11.7 years, body-mass index: 24.7 +/- 4.2) were wearing the Elan for up to three 24-hour periods during daily life [5]. In a study about atrial fibrillation detection, the Elan was worn for 24 hours during a Holter evaluation by patients suspected of having AF [6, 7].

Studies have also been executed where participants have been wearing the Elan for more than 24 hours. For example, for the EIT Digital project Fit-to-Perform, truck drivers (all males, 50 +/- 7 years old, 94% having a BMI > 25) were wearing the Elan for a duration of 2 days-1 week. In a study about core body temperature monitoring, 20 healthy participants, aged between 19 and 54 years and with a BMI of 19.6-26.8, have been wearing the Elan for 3 days in a row.
Even longer measurement periods have been studied more recently. At the end of last year (2018), 5 healthy participants (elite runners aged 18-35, BMI 17.8-20.5) have been wearing the Elan for 2 weeks, without complaints and a group of 10 healthy participants (age range 28-77 years old, average age 59 years old, BMI ranging from 20-28) have been wearing the Elan for 4 weeks consecutively. In the latter case, participants also used the MSX device at home to offload the data and charge the devices. All participants of this study were positive about their participation and they would participate again in the future. Two participants reported to have had some skin irritation during the wearing period. We believe that the above reviewed research shows that the Elan can be used in the current study.

4 RISKS AND BENEFITS ASSESSMENT

☒ I have created and uploaded a risk management summary matrix

☒ I have created and uploaded a risk management plan

A summary overview of the risks and benefits of the investigational device and the clinical procedure applied in this clinical investigation are listed in the table below.

**Anticipated clinical benefits**	For the patients participating in this investigation no benefits have been identified.<br><br>For future patients and the health system in general this study will deliver data and algorithms to assess deterioration continuously in an easy way for patients in hospital and at home.
**Anticipated adverse device effects**	Potential chemical, electrical, biological hazards associated with the devices have been identified, scored and if needed mitigated, see Risk Management Plan and Risk Management Summary Matrix of PJ-013483 FLAGSHIP Transitional Care Study 3, which are part of the study dossier and stored at the ICBE sharepoint. The conclusion from this Risk Analysis is that all evaluated risks are acceptable and that using this solution in an observational trial does not lead to an unacceptable risk for the patient.
**Residual risks associated with investigational device [as identified in risk analysis report]**	The residual risks associated with the devices and their potential interference with the clinical work flow have been identified, scored and mitigated. From the risk analysis it is concluded that the residual risks associated with the devices are acceptable, see Risk Management Plan and Risk Management Summary Matrix of PJ-013483 FLAGSHIP Transitional Care Study 3, which are part of the study dossier and stored at the ICBE sharepoint.

	The residual privacy risks associated with the study are that the devices with heart rate and respiratory rate data on it could come of and fall in the hands of unauthorized people.
**Risks associated with participation in clinical investigation**	No additional risks for participating in this investigation have been identified. Though the patients undergo surgery with the associated risks of this procedure this is not part of the study and the study does not influence the surgery risks. The placement of the devices will add some additional burden to the patient not related to her/his stay in hospital, however this additional burden is low.
**Possible interactions with concomitant medical treatments**	There are no interactions with concomitant medical treatments.
**Steps that will be taken to control or mitigate risks**	**Mitigations related to device risks** related to skin issues are:<br>1. Use of certified materials in contact with the skin.<br>2. Test of devices with healthy volunteers yielded highly favourable outcome on comfort and wearability.<br>3. Patient can take off the devices and stop participation.<br><br>**Mitigations related to privacy risk** are:<br>1. Data on the device will be encrypted.<br><br>The device will contain vital signs data but will not contain data that can be used to identify a patient (no; patient name, address, etc.).
**Risk-to-benefit rationale**	After re-evaluation of the risks, the overall residual risk has been deemed acceptable. As an overall result we conclude that due to the low burden and the acceptable risks it is justified to conduct this study.

5 OBJECTIVES AND HYPOTHESES

This chapter describes the objectives and hypotheses of the clinical investigation, as well as the claims and intended performance of the investigational device to be verified, and the risks and anticipated adverse device effects to be assessed.

Primary objective:

- To evaluate the sensitivity and specificity for the prediction of deterioration after surgery using the data calculated based on accelerometer and/or PPG measurements.

Secondary objective(s)

- To evaluate if the calculated heart rate and respiratory rate data are accurate compared to a gold standard as used in the hospital.
- To evaluate the usability of the devices from a hospital staff perspective not interfering with hospital workflow.
- To evaluate the usability of the HealthDot from a patient perspective not interfering with normal daily activities.

- To evaluate if connectivity is good during the patient journey and geolocation can be assessed.
- To evaluate if assessing baseline values of heart rate and respiratory rate adds value to the prediction of postoperative complications.
- To technically validate offline metrics using Elan collected data in the perioperative period as input (PPG and ACC) compared to those obtained from gold standard patient monitoring, which includes ECG (for heart rate), pulse oximetry, body temperature and invasive or non-invasive blood pressure measurements as part of standard care.

Primary hypothesis

- The calculated heart rate and respiratory rate from accelerometer measurements (Healthdot) and/or the metrics calculated from the PPG and accelerometer signals collected by the Elan could have predicted a deterioration of health in surgical patients. This hypothesis will be accepted or rejected based on the outcome of this clinical investigation.

Secondary hypotheses

- The calculated heart rate and respiratory rate of the HealthDot are comparable to the gold standard used in the at the hospital.
- The HealthDot is usable and does not interfere with the workflow of the hospital staff.
- The HealthDot is usable and does not interfere with normal daily activities of the patient in hospital and at home.
- Measuring heart rate and respiratory rate before surgery at home adds important information in predicting complications after surgery in the same patient.
- The offline metrics using Elan collected data in the perioperative period are comparable to those obtained from gold standard patient monitoring.

These hypotheses will be accepted or rejected based on the outcome of this clinical investigation.

Claims and intended performance
Since we use non medical devices and the study is not in full compliance to EN ISO 14155 no specific medical claims will be verified.

6 CLINICAL INVESTIGATION DESIGN

6. 1 General

This investigation is designed as a monocenter study at the Catharina hospital in the Netherlands. If more sites will be included during the conduct of the study amendments will be submitted to all respective internal and external approvers (ICBE, METC).

The primary endpoint is to evaluate the sensitivity and specificity for the prediction of deterioration after surgery using the data calculated based on accelerometer and/or PPG measurements.

The deterioration is primarily defined as complication according to Clavien Dindo classification grade II or higher. Complications are further assessed by the following events:

- Unplanned ICU admission
- Rapid Response Team (RRT) visit to patient
- Start of antibiotics
- Re-surgery
- Radiologic intervention such as abscess drainage

- Suppletion of erythrocytes, thrombocytes and Fresh Frozen Plasma
- Increase in early warning scores
- Readmission after discharge
- Death

The secondary endpoints of the study are:

- Agreement of the calculated heart rate and respiratory rate compared to the gold standard.
- Description of the extent of hampering in daily activities by both devices as assessed by the patients.

6.2 Investigational device(s) and comparator(s)

Within this study in some patients the calculated heart rate and respiratory rate of the HealthDot and the Elan device will be compared retrospectively to the hospital system for assessing vital signs in the recovery room and ICU after surgery. In the ICU the CARESCAPE Patient monitor B650 is used to collect and supervise vital signs. In the recovery room the Philips IntelliVue MP70is used to measure these data (see figure 5).

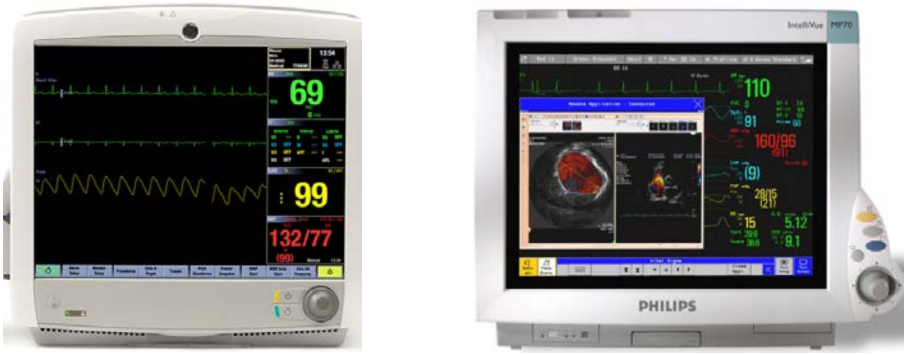

Figure 5: CARESCAPE Patient monitor B650 (left) used in the recovery room and Philips IntelliVue MP70 (right) used in the ICU.

These monitors measure a variety of vital signs that will be compared to the data gathered by the devices (HealthDot and Elan).

HealthDot:
The HealthDot will log an average HR and RR value every minute on the device. The HealthDot data will be sent to the backend with a timestamp every 5 minutes. The timestamps can be used to synchronize the HealthDot data with the gold standard data. The standard data are also averaged over the same period so a synchronization on a second base is not needed.

Elan:
From the Elan signal, we detect the timing of the individual heart beats. From the gold standard ECG measurements we do the same. Using the clock time of both the Elan and the ECG as a first approximation, we refine time alignment by maximizing the match between the detected heart beats in the two signals. This has been done in another study before.

6.3 Subjects

350 patients, scheduled for surgery e.g. bariatric and major surgery such as cytoreductive surgery and hyperthermic intraperitoneal chemotherapy (HIPEC), complex rectal surgery, esophagectomy and pancreatectomy.

Inclusion criteria for subject selection:

- Adult
- Willing and able to sign informed consent form
- Willingness to abstain from visiting a sauna during the study period
- Willingness to dry area where the HealthDot is applied in a dipping fashion after washing
- Willingness to abstain from flying during the study period of time
- Elective surgery
- General anesthesia required for surgery

Exclusion criteria for subject selection

- General inmates of psychiatric wards, prisons, or other state institutions
- Investigator or any other team member involved directly or indirectly in the conduct of the clinical study
- Any skin condition, for example prior rash, discoloration, scars or open wounds at the area of investigation of both devices
- Pregnant, or breastfeeding
- Known to be allergic for the tissue adhesive used in the HealthDot.
- Use of topical that is known to influence the skin at the test area (such as medical and non-medical creams or lotions)
- Patient with active implantables such as Implantable Cardioverter Defibrilator (ICD) and pacemaker
- Unable to understand instructions
- Expected participation less than 2 weeks
- Left lower rib (place where HealthDot will be applied) is involved in the area of surgery, area of disinfection or area where bandages are needed.
- Area on arm where the Elan device is applied is involved in the surgical procedure.
- Patients with antibiotic resistant infections (e.g. MRSA).

Criteria and procedures for subject withdrawal or discontinuation
Subjects can leave the study at any time for any reason if they wish to do so without any consequences. The investigator can decide to withdraw a subject from the study for medical reasons.

If one of the following criteria is fulfilled, the participation of a subject to the study is terminated:

- Withdrawal of informed consent
- Serious adverse events that were judged by the investigator to have a reasonable possibility that the event may have been caused by the investigational device
- Pregnancy
- Investigator’s decision for any safety reasons and/or medical judgment in the best interest of patients' health

Dropouts during screening (screening failures) and during the first 2 days within the study will be replaced.

The point of enrolment is the time at which, following recruitment, a subject signs and dates the informed consent form. The first subject is expected to be enrolled in April 2019. The Investigation is expected to take 15 months.
The duration of the participation of each subject is 3 weeks. For 20 subjects wearing the devices also before surgery for baseline measurement the time of participation will prolong by one week to up to 4 weeks (depending surgery schedule).

In this investigation, around 350 subjects will be enrolled. The enrolment period is planned to be April 2019 to June 2020.

6.4 Procedures

Specimen Banking (saving human fluid or tissue for future research)

☒ Not applicable—this is a human study without specimen banking.

6.4.1 Screening (Visit 0)

The patients are recruited during the pre-operative anesthesiology screening visit. Patients will be made aware of the study by their own doctor/surgeon. They will be asked if they would be interested to participate. If they consent, the investigators (different persons than the doctor/surgeon) will inform the patient about the study. Patients are informed about the experimental procedures and handed out the information letter including the informed consent form. The patients will have enough time to consider their participation. If the patient agrees to participate and signs informed consent she/he can be enrolled into the study.

6.4.2 Prebaseline visit (only 20 patients)

20 patients of the total of 350 planned patients, will be recruited to wear the HealthDot for up to 1 week and 2 Elans for 24 hours each before surgery to get their baseline values on heart rate and respiratory rate. The application of the devices will be done after patients signed informed consent. The patient will receive 2 Elan devices, one will already be placed on the wrist. After 24 hours the patient will switch of the first Elan and replace it with the second one. This will also be worn for 24 hours. The Healthdot will be removed by the patients themselves after 7 days. They need to bring the devices back to the hospital for their actual appointment for surgery.

6.4.3 Start (Baseline visit)

Patients who have agreed to participate in the study will come back to hospital for admission and surgery. After surgery the skin will be cleaned with a common alcohol wipe used in hospitals to make sure the HealthDot will stick well and then the HealthDot will be applied to the skin. In between cleaning and application 5 minutes waiting time will be used in order to allow the skin to dry off. The HealthDot will be applied in both male and female volunteers mid-clavicular on the lowest left rib of the chest. The HealthDot can be removed by the patient themselves in case of emergency with a remover tissue which will be handed out to the patient during the baseline visit. The patient will be instructed on how to remove the HealthDot with the tissue by the investigators. The Elan device will be applied at this time point too. The Elan will be applied at the wrist of the patient. After application of the HealthDot and Elan, the patients will continue to stay in hospital on discretion of the physician in line with standard care. Patients will be monitored on the Intensive care Unit or wake up room after surgery with the hospital monitoring system (see 6.2) and these data will be extracted and compared to the data gathered by the HD and Elan.

6.4.4 Discharge from hospital

Patients will be released from hospital on the discretion of the physician and return to their homes wearing the HealthDot and the Elan device. Each patient receives a second Elan for when the other

one is charging and an MSX device to charge and offload the Elan. They will be asked to wear an Elan for 24 hours for up to three weeks and to connect the Elan to the MSX device every evening and then wear the other Elan until the next evening.
When the patient is back at home the patient will be asked to fill in a diary each day with a few questions on paper (mini-diary 5-10min) for up to 2 weeks. This information will be collected at the end of the study, when the HealthDot (after wearing for 2 weeks in total) and Elan (after wearing for three weeks in total) will be removed. There are no questions included related to sensitive data. A sample of this diary can be found in Appendix I.

6.4.5 Follow up

All patients will remove the HealthDot and Elan themselves if they are at home or get the device removed when visiting the hospital for e.g. follow up exam. On this day, they will be asked to fill out a small additional questionnaire (15 min). A sample of this questionnaire can be found in Appendix I. All materials including the HealthDot, Elan and MSX device will be sent back by the patient to the hospital in a box provided by the investigator if they are at home or hand them in if they are visiting the hospital for e.g. follow up exam, In case a patient does not return the equipment, the patient will be contacted or visited.

Sponsor activities
All devices will be disinfected after return by the investigator and before return to Philips to avoid any possibility of infections by handling persons due to potential contamination by the patient.

6.5 Monitoring plan

Though this study is not fully compliant with ISO 14155 we will implement monitoring. This monitoring will be done risk based and since this is a low risk study three monitoring visits including initiation and close out visit are planned. Details of the monitoring process can be found in the monitoring plan [CT18_Monitoring_Plan_PJ-013483] which is part of the study documentation.

7 STATISTICAL CONSIDERATIONS

For baseline characteristics, results will be reported as mean (standard deviation) for normally distributed data and median (interquartile range) for data that are not normally distributed. Groups will be compared using the Students' t-test, Mann-Whitney test, Kruskal-Wallis test or Chi square test as appropriate. A p-value lower than 0.05 or adequate confidence intervals will be regarded as statistically significant. Non-parametric tests of comparison will be used for variables evaluated as not normally distributed. Linear regression models will be used to evaluate continuous data, and logistic regression models for categorical data. Data management and analysis will be performed using SPSS version 25.

Given the longitudinal nature of the study where various outcomes are measured repeatedly over time, this study allows addressing questions about changes over time in the response and the relationship between the response and subjects' characteristics. Data obtained from the HealthDot and Elan will be used to extract features such as average heart rate, interbeat interval (IBI), respiration rate, heart rate variability, activity type, motion level, motion frequency, orientation, and other time- and frequency-based motion features. The changes over time of these features can be modeled using (linear or nonlinear) mixed effects models [8]. The fitted models will be used to investigate whether subjects'

characteristics are related to changes in the longitudinal profile. By using joint models [9, 10], where the mixed model is combined with a time-to-event model, the association between changes in the longitudinal profile and a survival outcome can be tested.

Treatment of missing, unused or drop-out data
Every attempt will be made to have a complete follow-up of every patient. Nevertheless, in case of dropouts (lost to follow-up, withdrawal and missing follow-up) the reasons will be collected and reported. Dropouts during screening (screening failures) will not be included in the statistical evaluation of efficacy and safety. Dropout patients (including those who stop within the first 2 days of treatment) will be evaluated like any patient completing the study regularly, within the analysis populations they qualify for. In the statistical evaluation missing values will not be replaced/imputed.

Sample size
No formal sample size calculation has been performed. The sample size of 350 patients, as estimated by the Sponsor and the clinical site personnel, is based on the documented occurrence of complications after surgery, which are between 5 - 25% (depending on surgery performed). This will allow a retrospective analysis of up to 50 patients experiencing a deterioration of health after surgery. In case the number of 50 deteriorations is reached or is exceeded before 350 patients have been included, it will be considered if recruitment can be stopped.

Significance and power
Due to the exploratory nature of this study no formal power analysis has been performed for this study. All statistical results which can be tested with the appropriate test are considered significant when the probability value is equal or below 0.05.

Expected drop-out rates
The expected drop-out rate is 10% (35 patients in this sample). Patients dropping out during the first 2 days of the study will be replaced until the final 350 patients are included and have participated more than the 2 days defined for replacement. During the interim analysis after 75 patients it will be checked if the drop-out rates are as expected and if they are much higher, we will reconsider the replacement of patients dropping out.

Pass/fail criteria
If 20% or more of complications including readmission could have been detected retrospectively is defined as pass criteria.

Interim analysis
It is planned to do an interim analysis after 75 patients have completed the study to assess if the study procedures need to be adapted to the special needs of this heterogeneous patient population. A second interim analysis will be performed after 250 patients have finalized the study. This will allow a more precise assessment if the number of included patients is sufficient to prove or disprove the primary hypotheses and endpoint.

Termination of entire study on statistical grounds
N/A since data analysis is performed only retrospectively when study is closed out.

8 DATA MANAGEMENT

Data Collection

It is the responsibility of the Principle Investigator to ensure the quality of the data being collected by the CRF.

Required data will be recorded on electronic CRFs. Questionnaires and diaries will be filled out by the patients themselves. The draft versions of the questionnaires can be found in Appendix I.

The HealthDot data are collected on the sensor and forwarded to the cloud server. Data concerning the quality of the LoRa signal are generated by the LoRaWan network and forwarded to HSDP (Health Suite Digital Platform). No data from the device itself will be transferred to the investigator during the trial and will only be analyzed retrospectively, so that the collected vital signs are not actionable during the study.

The Elan data are collected on the device. At the hospital, data will be offloaded once a day by the investigator via USB onto the MSX device. At home, data will be offloaded once a day by the patient via USB onto the MSX device. The MSX device automatically uploads the data to the Barista platform (via 4G). Data that comes in during the course of the study will only be checked for completeness and correctness and no data analysis will take place until after the study is finalized.

In the hospital, data from the electronic medical record will be collected retrospectively for a dataset to analyse prediction models for complications in combination with the data from the HealthDot and Elan.

Data Validation/Handling
The data of the questionnaires and diaries will be entered in an Word/Excell document. The questionnaire and diary source data is locked in the eCRF (Research manager) system and is traceable and auditable. This document is not validated but since the pilot is not a clinical trial this is not a requirement.

Source Documents
The questionnaires and diaries will capture the data on adhesive strength and user experience and adverse events. The questionnaires and diaries will be the source.

Heart rate, respiratory rate, activity and posture data will be stored on the internal memory of the device (see section 2,3) and send via the network to the server. This data will be the source to which the received and stored data on the cloud server can be validated.

Subject Confidentiality
All information and data sent to all parties involved will be considered confidential. The study site will assign a unique subject ID number to each volunteer. Confidentiality of data will be observed by all parties involved at all times throughout the study. All data shall be secured against unauthorized access. The privacy of each subject shall be preserved in reports and when publishing any data.

Data relating to the study might be made available to third parties (for example in case of an audit performed by regulatory authorities), provided the data are treated as confidential and that the subject's privacy is guaranteed. No identifiable subject information will be published.

Data retention
The Investigator is responsible for retaining all study documents, including but not limited to the protocol, Investigator's Brochure and EC correspondence.
The site should plan on retaining study documents for at least 15 years after completion of the study. It is requested that at the completion of the required retention period, or should the Investigator retire or relocate, the Investigator contacts the Sponsor, allowing the Sponsor the option of permanently retaining the study records.

9 AMENDMENTS

If needed, the CIP can be amended according to the following procedure:
The request for amending a CIP may originate from various sources (sponsor, EC(s), investigator(s), etc). The final decision and responsibility lie with the sponsor.

For each change to the CIP, the amendment will include at least the following:

- Previous text
- Revised text
- Reason for amendment

A substantial amendment will also include all previously written non-substantial amendments, if applicable.

For substantial amendments Philips will ensure that each investigator signs and dates the amendment as a confirmation of compliance with the amendment. The original will be filed in the Trial Master File; a copy will be filed in the Investigator Site File.

For non-substantial amendments the sponsor will decide if it is required to obtain signatures from the PI. The original will be filed in the Trial Master File; a copy will be filed in the Investigator Site File.

The amendments to the CIP and the subject's informed consent form shall be notified to, or approved by, the EC and ICBE (see item 10 below). The version number and date of amendments shall be documented.

10 DEVIATIONS

Protocol deviations are any alteration or deviation from the ICBE-approved research plan as defined in the study protocol. This includes equipment failures during study procedures. The researcher will deviate from the protocol whenever necessary to protect the participant's health, rights or welfare and these types of deviations will be reported to ICBE and the external REC (if part of the study) as soon as possible. Major deviations must be reported to ICBE and the external METC (if part of the study) as soon as possible. In non-urgent/emergent situations, the researcher will obtain ICBE-approval for the planned deviation in advance of performing the changed activity.

If the researcher anticipates that there will be future requests for the same deviation, then the protocol will be amended (and such amendments must be approved by ICBE and the METC). A minor deviation is something that does not cause harm or have the potential to cause harm to the participant, and does not impact the integrity of the study. These shall be documented in the study file and uploaded to ICBE for their awareness. Other reasons for amendment requests include adding a study site, increasing the number of research participants, and extending the time to perform the study. If the study objectives and procedure or cohort changes or the study changes from non-medical to medical, this generates a new study (not an amendment).

Corrective and preventive actions and principal disqualification criteria
All CIP deviations will be documented and assessed. If required, corrective and preventive action will be agreed upon and implemented with the relevant site. These decisions, activities and possible preventive actions will be documented.

A Principal Investigator can be disqualified if he has repeatedly or deliberately failed to comply with the requirements as specified in the CIP, including compliance with the relevant regulations, or if he has submitted false information in any required report.

Corrective actions may include supplemental protocol training, discussions with PI and study staff for activities to prevent future recurrence, etc.. Misconduct can cause PI disqualification.

11 DEVICE ACCOUNTABILITY

The procedures for the accountability of investigational devices are in accordance with the Guidance. The access to investigational devices will be controlled and the investigational devices will only be used in the clinical investigation and according to the CIP.

The sponsor shall keep records to document the physical location of all investigational devices from shipment of investigational devices to the investigation sites until return or disposal. This record will be based on the device accountability log.

The principal investigator or an authorized designee shall keep records documenting the receipt, use, return and disposal of the investigational devices. This record shall include:

- the date of receipt,
- identification of each investigational device (batch number/serial number or unique code),
- the expiry date, if applicable,
- the date or dates of use,
- subject identification,
- date on which the investigational device was returned/explanted from subject, if applicable, and
- the date of return of unused, expired or malfunctioning investigational devices, if applicable

12 STATEMENTS OF COMPLIANCE

The investigation will be conducted according to the principles of the Declaration of Helsinki and other guidelines, regulations and Acts. The study will be conducted in accordance with the Medical Research Involving Human Subjects Act (WMO).

The investigation shall not begin until the required approval/favorable opinion from the EC or regulatory authority have been obtained and any additional requirements imposed by the EC or regulatory authority shall be followed.

Compensation for injury
The sponsor has a liability insurance in accordance with article 7, subsection 6 of the WMO.

The sponsor (also) has an insurance in accordance with the legal requirements in the Netherlands (Article 7 WMO and the Measure regarding Compulsory Insurance for Clinical Research in Humans of 23th June 2003). This insurance provides cover for damage to research subjects through injury or death caused by the study.

- € 650.000,-- (i.e. six hundred and fifty thousand Euro) for death or injury for each subject who participates in the Research;
- € 5000.000,-- (i.e. five million Euro) for death or injury for all subjects who participate in the Research;
- € 7.500.000,-- (i.e. seven million and five hundred thousend Euro) for the total damage incurred by the organization for all damage disclosed by scientific research for the Sponsor as 'verrichter' in the meaning of said Act in each year of insurance coverage.

The insurance applies to the damage that becomes apparent during the study or within 4 years after the end of the study.

13 INFORMED CONSENT PROCESS

Select all that apply:

The requirement for informed consent has been waived (i.e., not required by law OR waived by IRB/REC/METC). Upload waiver if available.

☒ Written informed consent will be obtained from adult participants

☐ Assent will be obtained from children/minors

☐ Parental permission will be obtained when children are participating

☐ Surrogate consent will be obtained when participants lack decisional capacity

☐ Deferred consent will be obtained [e.g., emergency research]

☐ Click-through consent will be obtained [Internet/app-based research]

☐ Implied consent [i.e., consent through action such as taking a test]

13.1 Consent Process

Study participation is voluntary. Potential subjects are given the most current ICBE/METC-approved consent form to read. They will be provided ample time for review and an opportunity to ask questions about the study. If they agree to participate, they will sign the consent form and be given a copy of the signed document for their records. Each of these actions/steps will be documented. Only after Informed Consent has been obtained, may the remaining study procedures begin.

13.2 New Information about the Study

Any new information about the study that may affect a consented subject's decision to be in the study (e.g., changed procedures, safety, etc.), will be communicated in a timely manner. Depending on the nature of the new information, subjects who have completed the study may or may not be informed, documenting the decision and justification as well as any activities for informing completed subjects. Additionally, the approving METC will also be informed. The currently approved Consent Form will be updated and submitted to the ICBE and approving METC for review and approval. Active subjects will be re-consented, following the above process, with the newly-approved consent form.

14 ADVERSE EVENTS AND DEVICE DEFICIENCIES

Definitions
Adverse events and adverse device effects, device deficiencies and serious adverse events and serious adverse effects and, unanticipated serious adverse device effects are defined in the table below:

Definition	***Description***
Adverse Event (AE)	Any untoward medical occurrence, unintended disease or injury, or untoward clinical signs (including abnormal laboratory findings) in subjects, users or other persons, whether or not related to the investigational medical device<br>NOTE 1 This definition includes events related to the investigational medical device or the comparator.<br>NOTE 2 This definition includes events related to the procedures involved.<br>NOTE 3 For users or other persons, this definition is restricted to events related to investigational medical devices.
Adverse Device Effect (ADE)	Adverse event related to the use of an investigational medical device<br>NOTE 1 This definition includes events related to the investigational medical device or the comparator.<br>NOTE 2 This definition includes events related to the procedures involved.<br>NOTE 3 For users or other persons, this definition is restricted to events related to investigational medical devices.
Serious Adverse Event (SAE)	Adverse event that<br>a) led to death,<br>b) led to serious deterioration in the health of the subject, that either resulted in<br>1) a life-threatening illness or injury, or<br>2) a permanent impairment of a body structure or a body function, or<br>3) in-patient or prolonged hospitalization, or<br>4) medical or surgical intervention to prevent life-threatening illness or injury or permanent impairment to a body structure or a body function,<br>c) led to foetal distress, foetal death or a congenital abnormality or birth defect<br><br>NOTE Planned hospitalization for a pre-existing condition, or a procedure required by the CIP, without serious<br>deterioration in health, is not considered a serious adverse event.
Serious Adverse Device Effect (SADE)	Adverse device effect that has resulted in any of the consequences characteristic of a serious adverse event or that might have led to any of these consequences if suitable action had not been taken or intervention had not been made or if circumstances had been less
Unanticipated Adverse Device Effect (UADE)	Serious adverse device effect which by its nature, incidence, severity or outcome has not been identified in the current version of the risk analysis report<br>NOTE Anticipated serious adverse device effect (ASADE) is an effect which by its nature, incidence, severity or outcome has been identified in the risk analysis report.

Reportability
All adverse events shall be reported to the Study Manager. ADEs, SAEs, SADEs and USADEs shall be reported to the Q&R office as well (Suzanne.Bloemsma@philips.com and

QandRoffice.pre@philips.com). The Director Q&R shall assess the ADEs, SAEs, SADEs and USADEs for reportability to Competent Authorities.

Reporting process
Reporting shall include the date of the adverse event, research subject ID#, description of the (serious) adverse event, treatment, resolution, and assessment of both the seriousness and the relationship to the investigational device and study procedures.

Timelines
The time period in which the principal investigator shall report all adverse events and device deficiencies to the sponsor and, where appropriate, to ECs and the regulatory authority, and the details of the process for reporting device deficiencies, are detailed in the table below.

Category	*Report to Study Manager*	*Report to Accredited Ethics Committee*	*Report to Q&R office* *QandRoffice.pre@philips.com* *Phone +31 40 27 95236 or +31 6 21459921* *Fax +31 40 274 6321 (inform us after you send a fax)*
	by *Principal Investigator*	*by* *Principal Investigator*	*by* *Principal Investigator* *by Study Manager*
Adverse Event (AE)	*Periodic reporting to Sponsor* *Periodic collection by Sponsor*	*As part of Sponsor's Clinical Investigation Report*	
Adverse Device Effect (ADE)	*Immediate, < 24 hr*	*As part of Ethics Committee Reporting timelines* *or* *< 24 hr following instructions by Sponsor*	*Immediate, <24 hr*
Serious Adverse Event (SAE)		*As part of Ethics Committee Reporting timelines* *or* *< 24 hr following instructions by Sponsor*	
Serious Adverse Device Effect (SADE)		*Immediate, < 48 hrs*	
Unanticipated Adverse Device Effect (UADE)		*As part of Ethics Committee Reporting timelines* *or* *< 24 hr following instructions by Sponsor*	

Emergency contact details

Joerg Liebmann
Philips Electronics B.V., Philips Research,
High Tech Campus 11
5656AE Eindhoven

•	**E mail**:	**During Business hours (09:00-17:00):** Joerg.liebmann@philips.com	
•	**Phone**:	**During Business hours** *(09:00-17:00)*: +31-(0)628498405	**Outside Business hours:** +31-(0)628498405

Foreseeable adverse events and anticipated adverse device effects
No anticipated adverse device effects have been identified. However, since the study described here aims at detecting deterioration of health in patients post surgery, the following adverse events not associated with this study but with surgery in general are expected:

- Postoperative ileus
- Delayed gastric emptying
- Anastomotic leakage
- Infection: wound, abdominal, pneumonia, urinary tract, central line infection
- Fistula
- Dehydration
- Bleeding
- Thrombosis: cerebral or cardiac infarction, intestinal ischemia, lung embolism
- Death due to any of the above mentioned postoperative complications (not associated with the study devices or study procedures)

In case of HIPEC

- Leucopenia
- Anemia
- Thrombopenia
- Heart, liver or renal toxicity

According to the Inspectie voor de Gezondheidszorg (Aanmeldingsformulier klinisch onderzoek met medisch hulpmiddel, Bijlage E 2017-07-14) these adverse events even if considered serious are not reportable since they are expected and described here as long as they do not lead to the suspension or termination of the trial or result in a change of the investigational product.

Information regarding the Data Management Committee, if established
N/A

Incidental Finding Reporting
An Incidental Finding is a finding concerning an individual research participant that has potential health or reproductive importance and is discovered in the course of conducting research but is beyond the aims of the study.

All incidental findings will be documented in a timely manner throughout the study. The report Incidental Finding Report Form (see ICBE SP) will be in the receipt of Philips Research Q&R Office within 7 business days of when the researcher first learns about the finding.

15 INVESTIGATOR BROCHURE (IB)

The Investigator Brochure (IB) is part of the study dossier and will be handed out to the investigator(s) before the study starts. This will be confirmed in writing.

16 VULNERABLE POPULATION

According to ISO 14155, a vulnerable subject is an individual whose willingness to volunteer in a clinical study could be unduly influenced by the expectation, whether justified or not, of benefits associated with participation or of retaliatory response from senior members of a hierarchy in case of refusal to participate.

☐ None—this study uses live human participants but not from vulnerable populations

Indicate whether you will include any of the following (vulnerable) populations in your study:

☐ Children or viable neonate (birth to age 28 days)

☐ Cognitively impaired

☐ Pregnant women (except USA)

☐ Fetuses

☐ Neonates of uncertain viability or nonviable

☐ Prisoners

☐ Poor/uninsured

☒ Educationally disadvantaged (limited education, e.g., high school drop out)

☒ Students (including interns, residents, fellows)

☒ Minorities (includes migrants, refugees)

☒ Elderly (over age 65 years)

☒ Terminally ill

☐ Other (check box and specifically discuss below)

In this study adult patients will participate including patients which are educationally disadvantaged students, minorities, older than 65 years and terminally ill only in case they are able to understand the study procedures and are able to provide informed consent themselves and none of the exclusion and all inclusion criteria are fulfilled. Therefore, these are not considered as vulnerable population for this study.

17 SUSPENSION OR PREMATURE TERMINATION

In case the study is ended prematurely, the investigator will notify the accredited EC and the ICBE about the reasons for the premature termination.

The principal investigator or authorized designee shall inform the subjects of the reasons for resumption.

The following criteria and arrangements for suspension or premature termination of the whole clinical investigation or of the clinical investigation in one or more investigation sites apply:
Early termination or suspension of the investigation will be considered if any of the following occur:

- Non-compliance to obtain patient informed consent
- Non-compliance to the inclusion/exclusion criteria
- Failure to follow patients per scheduled visits
- Failure to submit data in a timely manner
- METC suspension of the trial or of the center

If the study is terminated prematurely or suspended:

- Sponsor will promptly inform the clinical investigators of the termination or suspension together with the reasons why this decision was taken. They must also inform the regulatory authority(ies) (as stated by the applicable regulatory requirements).
- The EC will be promptly informed and provided with the reasons(s) for termination or suspension by the sponsor or by the clinical investigator.
- The investigator will promptly inform the patients and their personal physicians and assure appropriate therapy and follow-up for the patients.
- In case of early termination the investigator agreement will be terminated.

If the investigator (or EC) terminates or suspends the investigation without prior agreement of the Sponsor:

- The investigator will promptly inform Sponsor and the EC, and provide a detailed written explanation of the termination or suspension.
- The investigator will inform the institution (as stated by applicable regulatory requirements).
- The investigator will promptly inform the patients and their personal physicians and assure appropriate therapy and follow-up for the patients.
- The sponsor will inform the regulatory authority(ies) (as stated by applicable regulatory requirements).

18 PUBLICATION POLICY

It is planned to register this study in a public database like clinical trials gov or Nederland trial register. All manuscripts, abstracts, or other presentations will be reviewed by the Sponsor prior to release. The detailed publication strategy is described in the IMPULS 2 contract (Impuls 2 HC Flagship topic Peri-Operative_Final_extended_signed-by_all) between the Sponsor and the clinical site.

19 BIBLIOGRAPHY

1. Pearse RM, Moreno RP, Bauer P, Pelosi P, Metnitz P, Spies C, et al. Mortality after surgery in Europe: a 7 day cohort study. Lancet (London, England). 2012;380(9847):1059-65.
2. Subbe CP, Duller B, Bellomo R. Effect of an automated notification system for deteriorating ward patients on clinical outcomes. Critical care (London, England). 2017;21(1):52.
3. Ludikhuize J, Smorenburg SM, de Rooij SE, de Jonge E. Identification of deteriorating patients on general wards; measurement of vital parameters and potential effectiveness of the Modified Early Warning Score. Journal of critical care. 2012;27(4):424.e7-13.
4. Petit C, Bezemer R, Atallah L. A review of recent advances in data analytics for post-operative patient deterioration detection. Journal of clinical monitoring and computing. 2017.
5. Radha M, de Groot K, Rajani N, Wong CP, Kobold N, Vos V, Fonseca P, Mastellos N, Wark PA, Velthoven N, Haakma R, Aarts RM (accepted). Estimating blood pressure trends and the nocturnal dip from photoplethysmography.
6. Bonomi, A. G., Schipper, F., Eerikäinen, L. M., Margarito, J., van Dinther, R., Muesch, G., de Morree, H. M., Aarts, R. M., Babaeizadeh, S., McManus, D. D. & Dekker, L. R. C. (2018). Atrial fibrillation detection using a novel cardiac ambulatory monitor based on photo-plethysmography at the wrist. Journal of the American Heart Association. 7, 15, 17.
7. Eerikäinen, L. M., Bonomi, A. G., Schipper, F., Dekker, L. R. C., Vullings, R., de Morree, H. M. & Aarts, R. M. (2018). Comparison between electrocardiogram- and photoplethysmogram-derived features for atrial fibrillation detection in free-living conditions. Physiological Measurement. 39, 8.
8. Bates, Douglas, et al. "Fitting linear mixed-effects models using lme4." arXiv preprint arXiv:1406.5823 (2014).
9. Wulfsohn, Michael S., and Anastasios A. Tsiatis. "A joint model for survival and longitudinal data measured with error." Biometrics (1997): 330-339.
10. Rizopoulos, Dimitris. Joint models for longitudinal and time-to-event data: With applications in R. Chapman and Hall/CRC, 2012.

20 APPENDIX I

<u>Mini-diary for when the patient is back at home</u>

The questions will be focused on the usability/connectivity of the HealthDot and wellbeing.
Example of the questions:

- How do you feel today physically? (scale 1-5) [we want to scan the data if we could have predicted this feeling]
- Were you in contact with a healthcare provider on something related to the surgery? If so, could you describe with whom (role, e.g., cardiologist, GP, physiotherapist, etc.) and why? [we want to scan the data if we could have predicted this contact moment]

- Have you been outside home today for more than 2 hours? If yes, on which location and what kind of activity? Please indicate per morning, afternoon, evening. [we want to understand where the HealthDot has no connection]

Draft questionnaire after completion of the study Looking back on the entire workflow (in the hospital, after surgery and at home), how comfortable did you experience the wearing of the HealthDot?

To what extend did the HealthDot hamper you? (scale 1-5) and why?

Was it so severe that you wanted to take off the HealthDot? (Yes/No), why Yes/No?

Please describe why you wanted to remove the HealthDot? A) skin irritation B) uncomfortable in wearing C) hampering daily activities (e.g. showering, getting dressed) D) other (please explain) to what extent did the HealthDot lead to skin irritation? (scale 1-5)

How did you experience the HealthDot removal?

Do you have any additional comments?

21 APPENDIX II

MULTITECH
CREATE • CONNECT • COMMUNICATE

Declaration of Conformity

Product name: MTCAP-LEU1-868

Name and Address of Manufacturer:
Multi-Tech Systems, Inc.
2205 Woodale Drive
Mounds View, Minnesota 55112 USA

This declaration of conformity is issued under the sole responsibility of the manufacturer.

Object of Declaration: The MTCAP-LEU1-868 is a LTE / LoRa IoT Gateway.

The object of the declaration as described above is in conformity with the relevant European Union harmonized legislation:
Directive 2014/53/EU
Directive 2011/65/EU

The conformity of the essential requirements set out in Art. 3 of the 2014/53/EU has been demonstrated against the following harmonized standards:

EN 300 220-1 V3.1.1
EN 300 220-2 V3.1.1
EN 301 489-1 V2.2.0
EN 301 489-3 V2.1.1 (2017-3)
EN 301 489-52 V1.1.0
EN 301 511 V9.0.2
EN 301 908-1 V11.1.1
EN 301 908-2 V11.1.1
EN 301 908-13 V11.1.1
EN 60950-01:2006 + A11:2009 + A12:2011 + A1:2010 + A2:2013
EN 62311-2008

Place: Mounds View, MN USA

(Signature)

Date: 7/6/2018

Tim Gunn
(Full Name)

Director of Product Support / Certifications

Multi-Tech Systems, Inc. • 2205 Woodale Drive • Mounds View, Minnesota 55112 U.S.A.
(763) 785-3500 or (800) 328-9717 • Fax: (763) 785-9874 • www.multitech.com

[End of document]
